# Lipid Mediators and Human Leukemic Blasts

**DOI:** 10.1155/2011/389021

**Published:** 2010-10-10

**Authors:** Rémi Fiancette, Christelle Vincent-Fabert, Estelle Guerin, Franck Trimoreau, Yves Denizot

**Affiliations:** ^1^Centre National de la Recherche Scientifique, CNRS UMR 6101, Faculté de Médecine, Université de Limoges, 2 rue Dr. Marcland, 87025 Limoges, France; ^2^Laboratoire d'Hématologie, CHU Dupuytren, 87042 Limoges, France

## Abstract

Some of the most potent inflammatory mediators share a lipid origin. They regulate a wide spectrum of cellular processes including cell proliferation and apoptosis. However, the precise roles and ways (if any) in which these compounds impact the growth and apoptosis of leukemic blasts remain incompletely resolved. In spite of this, significant advances have been recently made. Here we briefly review the current knowledge about the production of lipid mediators (prostaglandins, leukotrienes, platelet-activating factor) by leukemic blasts, the enzymatic activities (phospholipase A_2_, cyclooxygenases, lipoxygenases) involved in their productions and their effects (through specific membrane bound receptors) on the growth, and apoptosis of leukemic blasts.

## 1. Introduction

**Figure 1:**
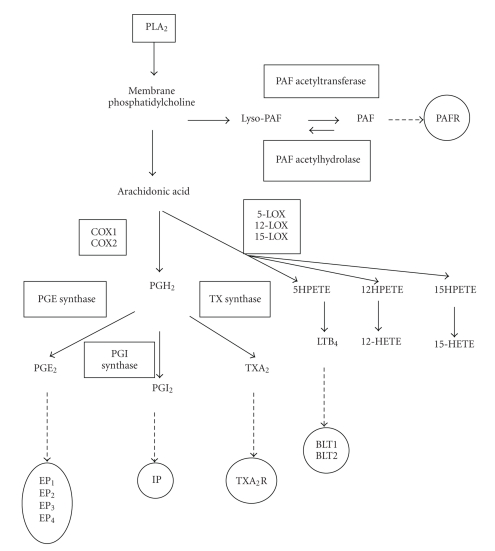
Simplified representation of the pathways involved in eicosanoid and platelet-activating factor formation and signal transduction. Enzymatic activities and receptors are in rectangles and ovals, respectively. PLA_2_, phospholipase A_2_; COX, cyclooxygenase; LOX, lipoxygenase; PGH_2_, prostaglandin H_2_; PGE_2_, prostaglandin E_2_; PGI_2_, prostacyclin; TXA_2_, thromboxane A_2_; HPETE, hydroperoxyeicosatetraenoic acid; LTB_4_, leukotriene B_4_; HETE, hydroxyeicosatetraenoic acid; PAF, platelet-activating factor; PAFR, PAF receptor; EP_1-4_, subtype 1–4 of the PGE_2_ receptor; IP, PGI_2_ receptor; TXA_2_R, TXA_2_ receptor; BLT_1-2_, subtype 1 and 2 of the LTB_4_ receptor.

Some of the most potent inflammatory mediators share a lipid origin. The action of phospholipase A_2_ (PLA_2_) on membrane phospholipids produces free fatty acids such as arachidonic acid (AA) and the phospholipid backbone. To the former belongs eicosanoids (such as prostaglandins, prostacyclin, thromboxane, and leukotrienes) through the cyclooxygenase (COX) and lipoxygenase (LOX) pathways; and to the latter, platelet-activating factor (PAF) (Figure 1) [1, 2]. While countless studies have highlighted the actions of eicosanoids and PAF on normal human mature myeloid and lymphoid cells (from hematopoietic progenitors to mature blood cells), their effects on leukemic blasts are poorly documented, and furthermore, their putative involvements during leukemic diseases remain almost speculative. This paper focuses on new results about lipid mediators and human leukemic blast cells from acute myeloid (AML) and acute lymphoid (ALL) patients. The vast majority of results reported previously have been obtained with AML blasts without maturation according to the classification system of the World Health Organization, thus corresponding to the past AML M_0-2_ nomenclature.

## 2. PLA_**2**_, PLA_**2**_ Receptors, and Human Leukemic Blasts

PLA_2_ catalyzes the hydrolysis of the *sn*-2 position of membrane glycerophospholipids to liberate the eicosanoid precursor AA (Figure 1) [3, 4]. Three distinct families are documented: low molecular weight soluble forms of PLA_2_ (sPLA_2_); Ca^2+^-dependent high molecular weight PLA_2_ (cytoplasmic PLA_2_, cPLA_2_); cytoplasmic Ca^2+^-independent high molecular weight PLA_2_ (iPLA_2_). In addition, the sPLA_2_ family is implicated in several biological processes such as inflammation and host defence [3, 4]. Nine isoenzymes have been identified. The cPLA_2_ family consists of four members, with cPLA_2_-IVA being the central regulator of the stimulus-coupled cellular AA release [3, 4]. The iPLA_2_ (PLA_2_-VI) plays a major role in phospholipid remodelling. Freshly isolated leukemic blasts from AML and ALL patients express mRNA from four out of five cPLA_2_ (PLA_2_-IVA, PLA_2_-IVB, PLA_2_-IVC, and PLA_2_-VI) and six out of nine sPLA_2_ (PLA_2_-IB, PLA_2_-IIA, PLA_2_-IID, PLA_2_-V, PLA_2_-X, and PLA_2_-XII) and that transcript levels exhibit wide variations as compared to control blood mononuclear cells [5]. One of the most notable findings is that AML and ALL blasts express high amounts of PLA_2_-VI and PLA_2_-X. This could be extremely significant as these two enzymatic activities play a major role in AA release for the generation of COX- and LOX-derived lipid mediators. Thus, AML and ALL blasts have the potential to express multiple isoforms of cPLA_2_ and sPLA_2_ which could be of importance given the role of these enzymes in inflammation, generation of lipid mediators, anticoagulant activity, and bacterial infection. Biological activities of PLA_2_ are attributed to their enzymatic capacity to hydrolyze membrane phospholipids. However, in addition sPLA_2_ exerts various biological proinflammatory responses through the binding to the cell surface PLA_2_ receptor (PLA_2_-R) [6]. Of interest is the functional membrane PLA_2_-R found on AML and ALL blasts strengthening a role for PLA_2_ signalling in these cells (Denizot and coll., in preparation). The concept of anti-inflammation is currently evolving with the discovery of endogenous inhibitory circuits, such as the annexin (ANX) system, that are important in the control of the host inflammatory response [7]. ANX-1 (also termed lipocortin) is a well-known cPLA_2 _inhibitory protein produced by and acting on several blood cell types such as monocytes/macrophages and polymorphonuclear leukocytes. The ANX-1 protein level is markedly elevated in AML blasts [8], where ANX-1 is not only considered as an anti-inflammatory and tumor suppressor molecule (through its inhibiting cPLA_2_ activity) but also as one of the “eat-me” signals on apoptotic cells to be recognised and ingested by phagocytes [8]. It is, thus, tempting to speculate that PLA_2_-R and ANX-1 might take an important place in the “yin” and the “yang” of the inflammatory reaction occurring in AML blasts. During the past decade, considerable research has been directed towards the identification of new biological targets for AML treatment. It is tempting to suggest that PLA_2_-R antagonists might be one of them especially with respect to the emerging roles for PLA_2_ enzymes in cancer [9].

## 3. COX and Human Leukemic Blasts

In the COX pathway, AA is converted to PGH_2_ by COX-1 or COX-2 enzymes. PGH_2_ is subsequently metabolised to generate different prostanoids, depending on the enzymes expressed in the cell [1]. The COX-1 isoform is typically constitutively expressed unlike the inducible COX-2 one. The growth-promoting properties of COX-2 in physiological responses are diverted in malignancies [10]. COX-1 and COX-2 transcripts are documented in AML and ALL blasts [11], but only the COX-1 protein is found. Similarly COX-1, but not the COX-2 protein, is detected in human primary promyelocytic blasts during differentiation [12]. In fact, the AML and ALL blasts can express the COX-2 protein in response to lipopolysaccharide (LPS) but only in the subsets of patients [13]. The ability of ALL blasts to express COX-2 is consistent with its presence in stimulated normal B-cells and in chronic lymphocytic leukaemia (CLL) B-cells [14, 15]. The production of COX-2 in response to LPS by AML blasts is consistent with data reporting that LPS is a potent inductor of COX-2 in mature monocytes/macrophages [16] and that stimulated HL-60 cells (an AML cell line with an M_2/3_ subtype) express COX-2 [17]. The heterogeneity in the LPS-stimulated COX-2 expression by AML blasts is not linked to a different Toll-like receptor (TLR2 or TLR4) expression [13] and remains an open question that requires further evaluation.

## 4. PGE_**2**_, EP Receptors, and Human Leukemic Blasts

Following the action of the COX pathway, PGH_2_ is subsequently metabolized to generate different prostanoids, depending on the enzymes expressed in the cell. Prostanoids include prostacyclin (PGI_2_), thromboxane A_2_ (TXA_2_), and prostaglandin E_2_ (PGE_2_), synthesized by a PGI_2_ synthase, a TXA synthase, and a PGE synthase, respectively [1]. Three PGE synthase isoforms exist: inducible membrane-bound PGE synthase-1 (mPGES-1), constitutive membrane-bound PGE synthase-2, and cytosolic PGE synthase. In addition, the ability of PGE_2_ to regulate the immune system has been widely explored [18]. Data reporting the ability of PGE_2_ to modulate several functions in mature blood cells such as monocyte-macrophages, dendritic cells, and T and B lymphocytes can be readily found. Human AML and ALL blasts spontaneously release PGE_2_ [11], with PGE_2_  synthesis being inhibited by indomethacin. Transcripts for mPGES-1 are detected in AML and ALL blasts suggesting its role in PGE_2_ synthesis (Denizot and coll., unpublished results). PGE_2_ effects are well known and are mediated through interactions with four distinct membrane-bound G-protein-coupled EP receptors: EP_1_, EP_2_, EP_3_, and EP_4_ [18]. EP_2_ and EP_4_ are coupled to G_s_ and stimulate cAMP production which leads to gene regulation. EP_3_ is coupled to G_i_ and inhibit cAMP synthesis. EP_1_ is coupled to G_q/p_, and ligand binding induces intracellular calcium level variations. Functional EP_2_ receptors are present on AML and ALL blasts [19, 20]. In contrast to EP_2_ receptors, no functional EP_1_, EP_3_, and EP_4_ receptors are found [20]. In view of the potentially important role of PGE_2_ in processes of cancer and leukocyte maturation and function, PGE_2_ effects have been investigated on blast cell proliferation and apoptosis. PGE_2_ enhances the spontaneous and LPS-stimulated growth of AML blasts without affecting their apoptosis [11]. In summary, AML and ALL blasts secrete PGE_2_. A role for PGE_2_ as a compound contributing to AML cell proliferation (via an EP_2_ receptor-mediated pathway) can be hypothesized.

## 5. TXA_**2**_, PGI_**2**_, and Human Leukemic Blasts

TXA_2_ and PGI_2_ are two other potent COX metabolites. TXA_2_ is produced abundantly by platelets upon exposure to injured blood vessels and thus exhibits potent platelet-aggregating and vessel-contracting activities. PGI_2_ is the major COX-derived product of AA formed in the macrovascular endothelium and is a potent inhibitor of platelet aggregation activity and vessel vasodilatation activity [21]. AML and ALL blasts express low levels of TX synthase transcripts compared to normal blood mononuclear cells (Denizot and coll., unpublished results) and additionally produce very low amounts of TXA_2_ in response to a calcium ionophore stimulation [22]. HL-60 cells have also been shown to release TXA_2_, but only after induction of differentiation [23, 24]. PGI synthase transcripts are absent in AML and ALL blasts, a result similar to that found in control blood mononuclear cells (Denizot et coll., unpublished results). In accordance with the absence of PGI transcripts in AML and ALL blasts, calcium ionophore-stimulated blasts do not release PGI_2_ (Denizot et coll., unpublished results). TXA_2_ and PGI_2_ act through membrane receptors (namely TXA_2_R and IP for TXA_2_ and PGI_2_, resp.) [25, 26]. As to whether AML and ALL blasts release TXA_2_ and PGI_2_, they express levels of transcripts for TXA_2_R and IP equal or higher than those found in control blood mononuclear cells [27]. TXA_2_R and IP receptors belong to the class of Gs-protein-coupled receptors [25, 26]. Stimulation of leukemic blasts with U-46619, the TXA_2_ receptor agonist U-46619, and PGI_2_ stimulate in a dose-dependant manner cAMP synthesis from leukemic blasts showing the presence of functional TXA_2_R and IP receptors, respectively [27]. However, simulation of leukemic blast with U-46619 and PGI_2_ has no effect on their growth and apoptosis rate. At the present time the physiological meaning of functional TXA_2_R and IP receptors on leukemic blasts remains an open question. In conclusion, among the various COX-derived metabolites of AA only PGE_2_ has, thus, a significant effect on the growth of AML blast cells [11], and none of them affect their apoptosis rate.

## 6. LOX and Human Leukemic Blasts

The LOX pathway involves the conversion of AA to 5-, 12-, or 15-hydroperoxyeicosatetraenoic acids (HPETE) by 5-, 12-, or 15-LOX, respectively, HPETEs being rapidly metabolized to 5-, 12, or 15-hydroxyeicosatetraenoic acids (HETE). 5-HPETE could be dehydrated into leukotriene A4 (LTA_4_), which was enzymatically hydrolyzed to LTB_4_ (Figure 1) [1]. The ability of LTB_4_, 12-HETE, and 15-HETE to regulate important functions of the immune system has been widely explored. These compounds activate various blood cell types and stimulate their proinflammatory cytokine productions [28–30], indicating an ability of LTB_4_, 12-HETE, and 15-HETE to augment and prolong tissue inflammation. Leukemic blasts express 5-LOX, 12-LOX, and 15-LOX transcripts, their expression being in general lower than in blood mononuclear cells from a healthy donor [22, 31, 32]. Leukemic blasts produce *in vitro* lower amounts of LTB_4_ than healthy donors [22, 31, 33]. This reduced capacity of AML blasts to produce LTB_4_ is located at the 5-LOX level. Stimulated leukemic blasts produce 12-HETE but not 15-HETE [22]. The various LOX-derived metabolites of AA regulate a wide spectrum of cellular processes including cell proliferation and apoptosis. 12-HETE and 15-HETE stimulate the proliferation and differentiation of normal CD34^+^ cells [34]. LTB_4_ induces proliferation and exerts an antiapoptotic effect on blood CD34^+^ cells [35]. However, LTB_4_, 12-HETE, and 15 HETE have no effect on the growth and apoptosis rate of AML and ALL blasts *in vitro* [22]. As to whether receptors for 12-HETE and 15-HETE remain to be molecularly identified, two G-protein-coupled seven transmembrane domain receptors for LTB_4_ were identified: BLT1 and BLT2 [36]. Amounts of BLT1 transcripts are similar in AML and ALL blasts as well as control blood mononuclear cells, while amounts of BLT2 transcripts are markedly higher [22]. At this time the physiological meaning (if any) of BLT1 and BLT2 transcripts in AML and ALL blasts remains an open question. A similar question exists for the significance of LTB_4_- and 12-HETE-derived leukemic blasts. One might suggest that these compounds could initiate, augment, and prolong tissue inflammation and damages by affecting the cytokine network, but currently no studies have provided evidences in support of this.

## 7. PAF, PAFR, and Human Leukemic Blasts

PAF is a phospholipid mediator that sparks off a wide range of immunoregulatory activities on blood cells such as polymorphonuclear neutrophils, eosinophils, monocytes, macrophages, and lymphocytes [2]. PAF is released *in vitro* from several leukemic cell lines of B and T origin [37] as well as from freshly isolated neoplastic cells of leukemic patients [38]. However, in spite of experimental evidence reporting its *in vitro* release from leukemic cells, no clinical studies provide evidences to support this view *in vivo*. In contrast, decreased levels of PAF are found in the blood of patients with lymphoid and nonlymphoid hematologic malignancies [39]. Blood PAF levels are regulated by an acetylhydrolase activity (AHA, also named PLA_2_-VIIA) found in serum and plasma. Plasma AHA is not altered in leukemic patients [39] suggesting a lowered PAF production by leukemic cells rather than an increased PAF catabolism. PAF acts through membrane and nuclear PAF receptors (PAFR) that belong to the G-protein-coupled family [40, 41]. As to whether membrane PAFR is found on AML and ALL cells [42, 43], intracellular ones were detected [42]. Studies report that mature monocytes, macrophages, polymorphonuclear leukocytes, and B lymphocytes produce cAMP in response to PAF [44, 45]. This is not the case for AML and ALL blasts [46]. PAF modulates Ca^2+^ flux through a Gq-protein-mediated pathway [47]. The Gq proteins mediate their effects by activating phospholipase C and thus, generating second messengers, inositol-1,4,5-triphosphate (IP3) and diacylglycerol, thereby leading to the activation of protein kinase C and the mobilisation of intracellular calcium. PAF stimulates in a receptor-dependent process Ca^2+^ flux from AML and ALL blasts showing the presence of functional PAFR [48] and highlighting that PAFR signals via the Gq instead of the Gi/Gs protein pathways. Hence, the role of PAF in leukemic blasts still remains an open question. PAF has no significant effect on growth and apoptosis rate in these cells [49] suggesting that PAF is not an important modulator of blast cell physiology. The lack of PAF effect is linked to low levels of PAFR in AML and ALL blasts compared to those found in mature leukocytes [49]. Further, strengthening this issue, the differentiation of HL-60 cells towards the macrophage phenotype is associated with the induction of PAFR gene expression. Thus, PAFR mRNA accumulation is correlated to the induction and development of specific PAF responsiveness [50]. Recently WEB-2170, a PAFR antagonist, has been reported to induce apoptosis in AML cells [51, 52]. In fact, WEB-2170 does not behave as a pure PAFR but instead as an inverse agonist leading to a marked cytoplasmic increase of PTEN proteins (PTEN is a protein/phosphoinositide phosphatase regulating the PI3K/Akt signaling pathway). Consequently, these recent results [49, 51, 52] support the view that PAF has probably no significant role in the growth and apoptosis of leukemic blasts.

## 8. Conclusion

**Figure 2:**
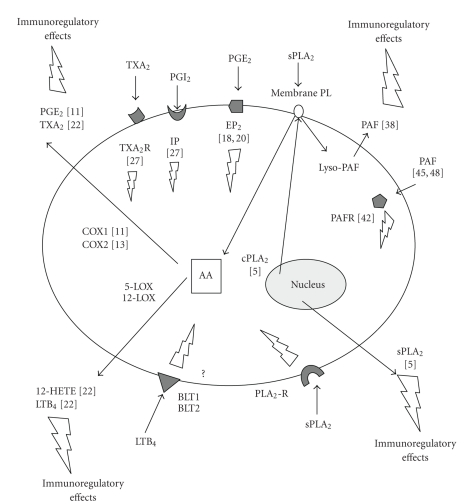
Simplified representation of the relationships between lipid mediators and leukemic blasts. Leukemic cells express several cPLA_2_ and sPLA_2_. COX activities can metabolise AA into PGE_2_ and TXA_2_. LOX activities can metabolise AA into LTB_4_ and 12-HETE. Leukemic cells can release PAF. Functional TXA_2_, IP, EP_2_, PAF, and PLA_2_ receptors are found on leukemic cells. BLT1 and BLT2 transcripts are detected suggesting (?) LTB_4_ receptors. The immunoregulatory effects of lipid mediators are currently speculative except for the role of PGE_2_ on AML blast growth. Related references are in square brackets.

Data reporting our knowledge concerning the enzymatic activities (such as PLA_2_, LOX, COX) implicated in lipid mediator synthesis and their receptors on AML and ALL blasts are schematised in Figure 2. Aberrant expression of several PLA_2_ enzymes is common place in tumors derived from many different organ sites [9]. Numerous studies report that altered AA metabolism in a solid tumor microenvironment has a profound impact on the pathogenesis of tumor development [1]. A multitude of biological activities of PAF are evidenced both on the normal cell as well as on their cancer counterpart [2]. There is evidence, however, that it is not the case for leukemic blast cells. Among the various pro-inflammatory lipid molecules so far tested (PAF, PGE_2_, PGI_2_, TXA_2_, LTB_4_, 12-HETE, 15-HETE), none of them exhibit any role on leukemic blast apoptosis despite the expression of functional receptors (PAFR, EP_2_, IP, TXA_2_R). Among the various compounds so far tested only PGE_2_ clearly demonstrated a potential role in AML cell growth *in vitro*. However, it is difficult to compare the *μ*M amounts of PGE_2_ used in most of the *in vitro* studies with the fM amounts of PGE_2_ found in the blood at steady state conditions. Studies showing the effects of continuous addition or infusion of low doses of PGE_2_ (which seems to be a more relevant protocol of stimulation to obtain information for the *in vivo *effects of PGE_2_) are extremely rare. Moreover, data obtained *in vivo* and *in vitro* are sometimes discordant. In fact, there is absolutely no evidence that PGE_2_ is implicated in the growth of AML blasts *in vivo*. Thus, in conclusion the biological effects of eicosanoid and PAF are particularly important in immunity and inflammation. Though their roles are well known in numerous pathology and cancers, no such role is currently known for leukemic blast growth.
